# Finding Inhibitors of Mutant Superoxide Dismutase-1 for Amyotrophic Lateral Sclerosis Therapy from Traditional Chinese Medicine

**DOI:** 10.1155/2014/156276

**Published:** 2014-05-18

**Authors:** Hung-Jin Huang, Tung-Ti Chang, Hsin-Yi Chen, Calvin Yu-Chian Chen

**Affiliations:** ^1^Department of Chinese Pharmaceutical Sciences and Chinese Medicine Resources, College of Pharmacy, China Medical University, Taichung 40402, Taiwan; ^2^School of Chinese Medicine, College of Chinese Medicine, China Medical University, Taichung 40402, Taiwan; ^3^Department of Biomedical Informatics, Asia University, Taichung 41354, Taiwan; ^4^School of Medicine, College of Medicine, China Medical University, Taichung 40402, Taiwan

## Abstract

Superoxide dismutase type 1 (SOD1) mutations cause protein aggregation and decrease protein stability, which are linked to amyotrophic lateral sclerosis (ALS) disease. This research utilizes the world's largest traditional Chinese medicine (TCM) database to search novel inhibitors of mutant SOD1, and molecular dynamics (MD) simulations were used to analyze the stability of protein that interacted with docked ligands. Docking results show that hesperidin and 2,3,5,4′-tetrahydroxystilbene-2-O-**β**-D-glucoside (THSG) have high affinity to mutant SOD1 and then dopamine. For MD simulation analysis, hesperidin and THSG displayed similar value of RMSD with dopamine, and the migration analysis reveals stable fluctuation at the end of MD simulation time. Interestingly, distance between the protein and ligand has distinct difference, and hesperidin changes the position from initial binding site to the other place. In flexibility of residues analysis, the secondary structure among all complexes does not change, indicating that the structure are not affect ligand binding. The binding poses of hesperidin and THSG are similar to dopamine after molecular simulation. Our result indicated that hesperidin and THSG might be potential lead compound to design inhibitors of mutant SOD1 for ALS therapy.

## 1. Introduction

Mutations in Cu/Zn-binding superoxide dismutase type 1 (SOD1) could decrease protein stability and increase aggregation; SOD1 variants are associated with amyotrophic lateral sclerosis (ALS) [1–4]. ALS belongs to motor neuron degenerative disease in the cortex, brainstem, and spinal cord [5, 6], which is similar to Alzheimer's disease, Parkinson's disease, and Huntington's disease, and the symptoms of ALS include degenerative disorder of upper/lower motor neurons and denervation of muscle fibres, leading to loss of motor neuron, progressive muscular paralysis, and muscle atrophy [7, 8], resulting in weakness of voluntary muscles till death because of respiratory failure [9]. A recent study indicated that mutation of SOD1 gene has genetic linkage in ALS disease, ALS-associated SOD 1 mutations including alanine 4 by valine (A4V) [10–13], histidine 46 by arginine (H46R) [14–16], and I113T mutation [17–19]. The most popular ALS-causing mutation is A4V mutant type in United States; near 50% of SOD1-ALS patients are associated with the A4V mutation [20]. H46R has been identified in Japanese, about 80% Japanese familial ALS in the affected members [21]. I113T type mutation is another one of the common SOD1 mutations of FALS [22], and many cases of clinical manifestations are linked to the I113T SOD1 mutation [23]. They proposed a toxic gain of function caused by mutant SOD due to the aggregation [24, 25]; hence, designing novel drugs for inhibition of SOD1 aggregation and stabilization have been used in ALS treatments [26].

The aim of this study is to focus on drug design of mutant SOD1. The complex of mutant SOD1 and dopamine was used to investigate the novel inhibitors of mutant SOD1 for inhibiting aggregation. Computer-aided drug design (CADD) is rapid approach for drug discovery [27–30], which is based on risk factor studies [31–36], theory [37], and web server [38] for developing novel leading compounds. CADD has been wildly used to design new drugs in many cases, such as virus [39, 40], inflammation [41], cancer [42–45], insomnia [46], weight loss [47], erectile dysfunction [48–50], nerve system [51–53], and diabetes [54]. Traditional Chinese medicine (TCM) has been used over two thousand years in clinical therapy, and many studies utilized TCM to investigate new therapies [55–58]. In our study, small molecules from the world TCM database [59] was used to screen for searching potential compounds with high affinity in mutant SOD1 active site, and we further utilized molecular dynamics (MD) simulation to verify the stability between protein and ligands for binding assay. Synthetic drug often has side effect in clinical treatments, and our results provided nature product as drug candidate, which is safer and reduce adverse reactions.

## 2. Materials and Methods

### 2.1. Database Screening

The crystal structure of mutant SOD1 was obtained from PDB database (PDB code: 4A7V) [60]. We employed* Prepare Protein module* of Accelrys Discovery Studio 2.5.5.9350 (DS 2.5) software [61] to clean up mistakes of each residue on mutant SOD structure, such as deleting alternate conformations, modeling missing loops, and removing water molecules. This module also predicts titration site pKs for each amino acid, and the pH value of 7.4 was used to protonate all residues. PONDR-FIT [62] was used to predict the order/disorder in mutant SOD1 structure. The 61000 TCM compounds were downloaded from the TCM Database@Taiwan [59] for database screening, we also employed TCM compounds from Chang' lab for binding assay [63], and MM2 force field [64] of ChemBioOffice 2010 software was carried out to optimize and calculate the 3D conformation of TCM compounds. All compounds generate different conformations by Monte-Carlo techniques under* LigandFit module* [65] of DS 2.5, which were docked into mutations SOD1 binding site for protein-ligand interaction analysis. Minimization of all docking poses was based on CHARMm force field [66], and we used Smart minimizer algorithm as minimization algorithm for ligands minimization [67, 68], which contains steepest descent and conjugate gradient. The steepest descent performed 1,000 steps and followed by conjugate gradient minimization.

### 2.2. Molecular Dynamics (MD) Simulation

Protein-ligand structures were obtained from results of docking study, and the starting conformation of protein-ligand complex was performed using GROMACS 4.5.5 package [69] for molecular dynamic simulation, using charmm27 force field. The protein structure was placed in cubic box containing TIP3P water molecules. The distance between protein and box was set to 1.2 nm, and the van der Waals cutoff to 1.4 nm. Particle mesh Ewald (PME) method is regard as coulomb type for calculating electrostatic interaction, and LINCS algorithm was used to restrain the lengths of all bonds among all simulations. For obtaining topology file and parameters of small compounds, we employed SwissParam to generate these data and compatible with the CHARMM all atoms force field for GROMACS simulation. In system neutralization, we added Na and Cl ions to randomly replace solvent molecules in simulation systems, and the concentration of NaCl model was set as 0.145 M. The time step was set to 0.002 ps for MD simulation. Steepest descent algorithm was applied to energy minimization for 5,000 cycle steps. The following procedure is equilibration, which was performed under position restraints for 100 ps to relax solvent in protein structure under constant temperature dynamics (NVT) condition. Production simulations perform 5000 ps at final step for all simulation systems under constant pressure and temperature (NPT) dynamics. Temperature of all simulation systems was set to 310 K. All MD frames were saved every 20 ps for trajectory analysis.

### 2.3. Analysis of MD Simulation

Trajectory analysis of MD conformations was calculated by GROMACS 4.5.5 [69], including root mean square deviation (RMSD), root mean square fluctuation (RMSF), and mean square displacement (MSD). The secondary structures analysis was performed by DSSP program under GROMACS 4.5.5. Linkage clustering algorithm was used to identify the most populated structural representations of conformation during MD simulations. The RMSD cutoff for cluster analysis was set as 0.13.

## 3. Results and Discussion

### 3.1. Docking Results of Database Screening

**Figure 1:**
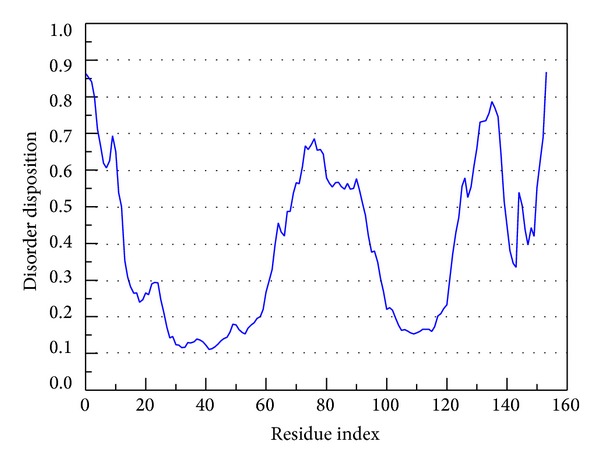
Disorder analysis of sequence of mutant SOD1 from result of PONDR-FIT prediction. The value of disorder disposition above 0.5 in disorder disposition is indicated as disorder residues.

**Figure 2:**
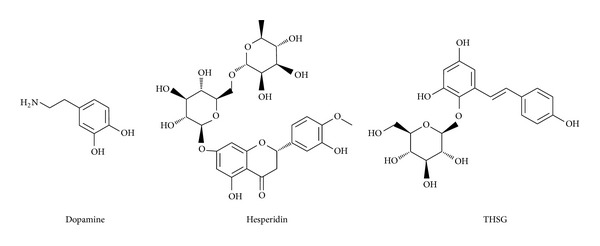
Chemical scaffold of dopamine, hesperidin, and THSG.

**Figure 3:**
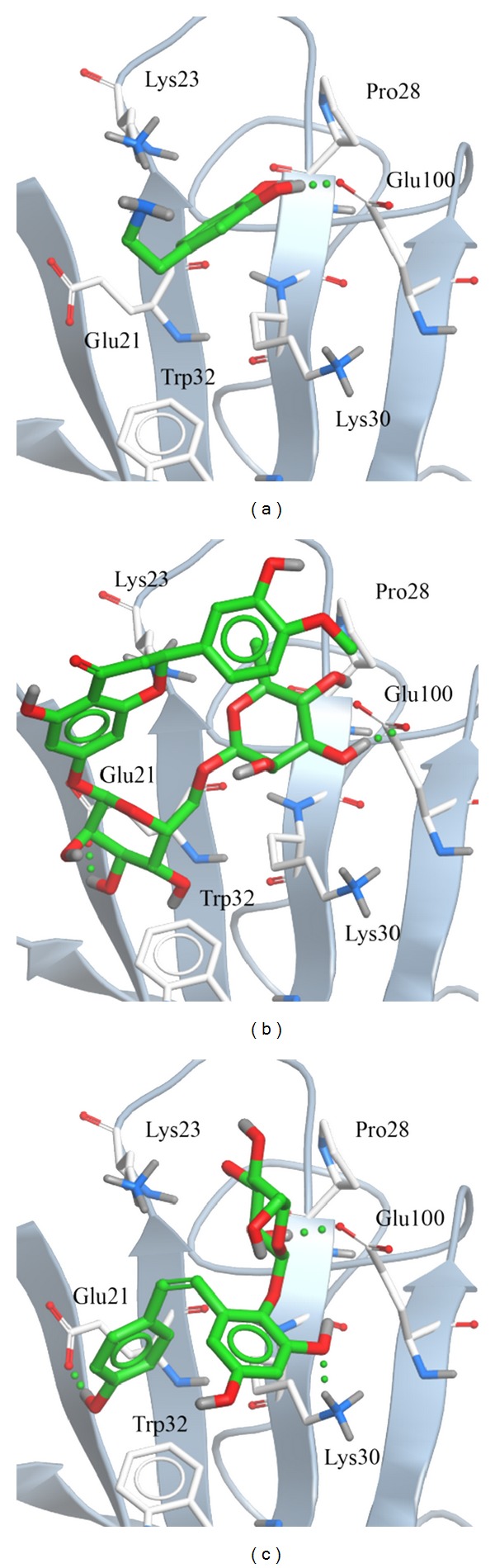
Binding poses of docking result: (a) dopamine, (b) hesperidin, and (c) THSG.

To analysis disorder region, we employed PONDR-FIT [62] to predict the order/disorder in mutant SOD1 structure. The sequence number from 21 to 32 and from 98 to 100 are binding site of mutant SOD1 (Figure 1). The disorder disposition values among this range are below 0.5, which indicates that the binding site is folded orderly and that the protein structure may not affect ligand binding [70, 71]. For docking analysis, we based on -PLP1, -PLP2, -PMF, and Dock Score to evaluate the docking pose of traditional Chinese medicine (TCM) compounds. From scoring analysis, dopamine was regarded as control for comparing with TCM compounds. The score values from docking poses of TCM compounds are shown in Table 1. All docked ligands are ranked by Dock Score, and we found that hesperidin and 2,3,5,4′-tetrahydroxystilbene-2-O-*β*-D-glucoside (THSG) [63] with Dock Score (including score values of -PLP1, -PLP2, and -PMF) are higher than dopamine. We selected hesperidin, THSG and dopamine for further studies, and chemical scaffolds of these small molecular are shown in Figure 2. Docking poses of dopamine, which displayed H bond with Glu100, the surrounding residues include Lys30, Lys23, Glu21, Pro28, and Gln22 (Figure 3(a)). For hesperidin, there are three amino acids (Glu21 and Glu100) generated H-bond interaction, and the surrounding residues are Trp32, Pro28, and Lys30 (Figure 3(b)). THSG has two amino acids generated H-bond for ligand binding, which are Glu21, Lys30 and Glu100, and amino acids that include Lys23, Pro28, and Trp32 are near the docked ligand (Figure 3(c)). It is worth to know that Glu100 is the common residue for each ligand binding, and the Lys30 can be found in all binding residues of mutant SOD1. In further study, we utilized molecular dynamics simulation to analyze variation of each ligand in protein structure.

### 3.2. Stability Analysis of Molecular Dynamics Simulation

**Figure 4:**
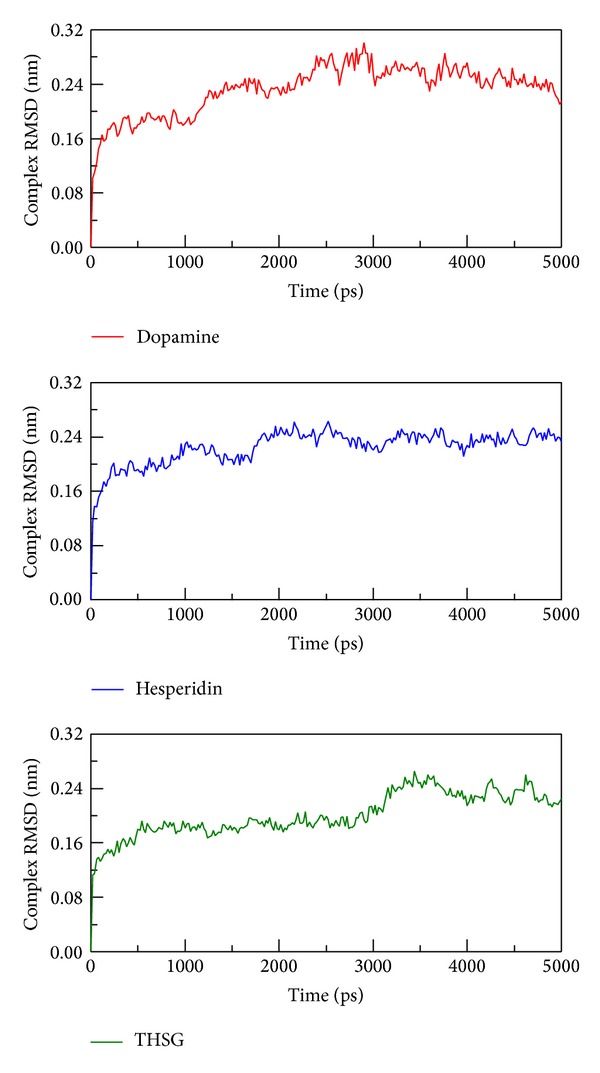
RMSD values of complex structures with docked ligand: dopamine, hesperidin, and THSG among 5000 ps simulation.

**Figure 5:**
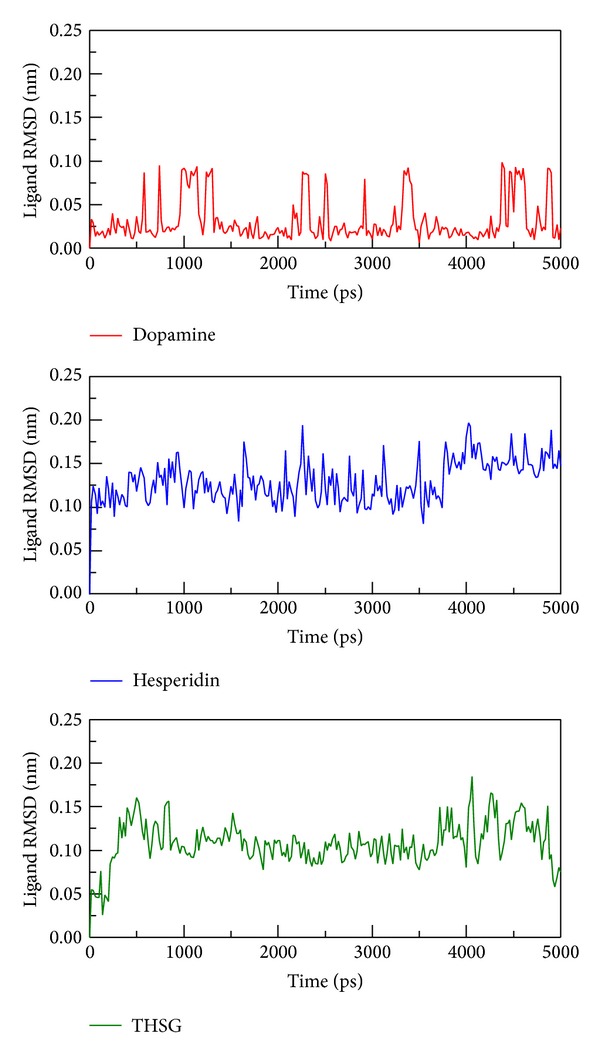
RMSD values of three compounds: dopamine, hesperidin, and THSG in protein complex during 5000 ps simulation time.

To determine stability of conformations among MD simulations, we utilized root mean square deviation (RMSD), radius of protein gyration, and total energy to analyze deviation of all complexes with docked ligand. The RMSD values of protein structure were used to verify stability among MD simulations.  Figure 4 shows all values within the range from 0.16 to 0.24 nm, which indicate that all protein structures from protein-ligand complexes are stable during 5000 ps simulation, and the 5000 ps simulation time is enough for decreasing the fluctuation of all complexes. For ligand RMSD (Figure 5), it is obvious that the conformation of dopamine displayed high degree of difference during dynamic simulation, and the value of ligand RMAD increased to 0.24 nm from 3000 ps to the end. Ligand RMSD of TCM candidates shows slightly deviation, and the values of hesperidin and THSG are in average of 0.15 and 0.10, respectively.

### 3.3. Migration Analysis of Molecules

**Figure 6:**
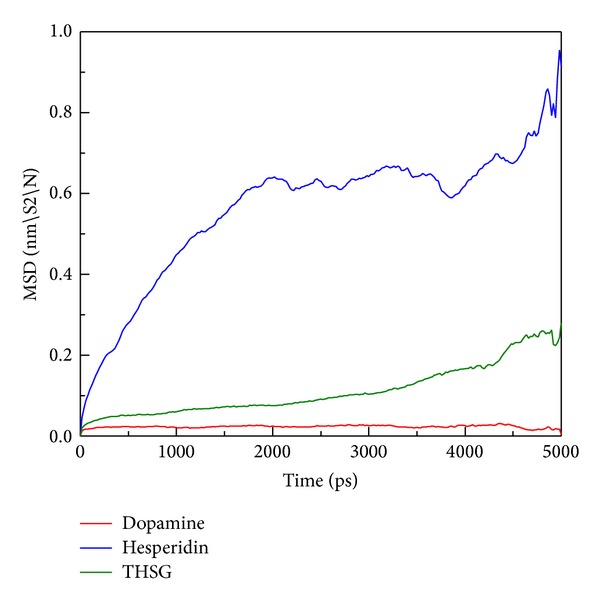
MSD values of three compounds: dopamine, hesperidin, and THSG in protein complex during 5000 ps simulation time.

**Figure 7:**
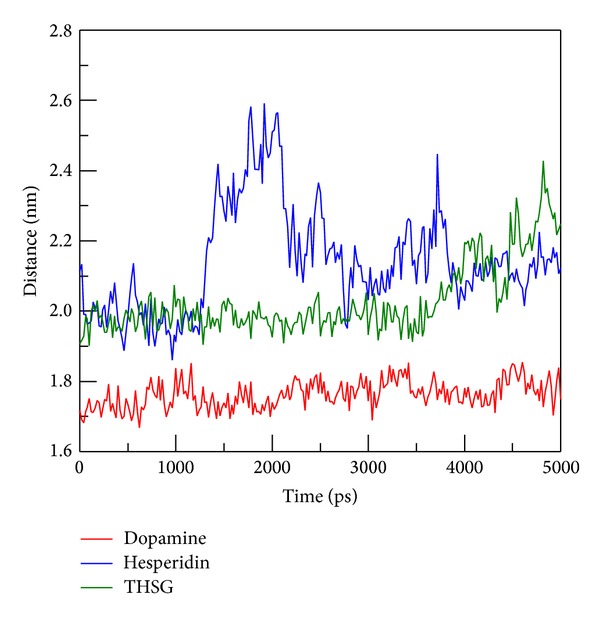
Distance between the centers of mass of mutant SOD1 and ligands.

In order to assess the variation of each ligand after being docked into protein binding site, MSD analysis was used to measure the migration of docked ligand during MD simulation. MSD value of hesperidin is the most distinct from the other TCM candidates, which displayed a rapid increase during initial simulation to the end of 5000 ps (Figure 6). In further study, we measured the distance between centers mass of protein and each ligand among all simulation times to understand movement of docked compounds. Interestingly, hesperidin shows a long distance with 2.6 nm (Figure 7) and turned to 2.0 nm after 2000 ps. Indicating that hesperidin moved away from the initial binding position and transferred to another site of the protein structure. The results suggest that each ligand could bind with mutant SOD1 during 5000 ps.

### 3.4. Flexibility of Residues Analysis

**Figure 8:**
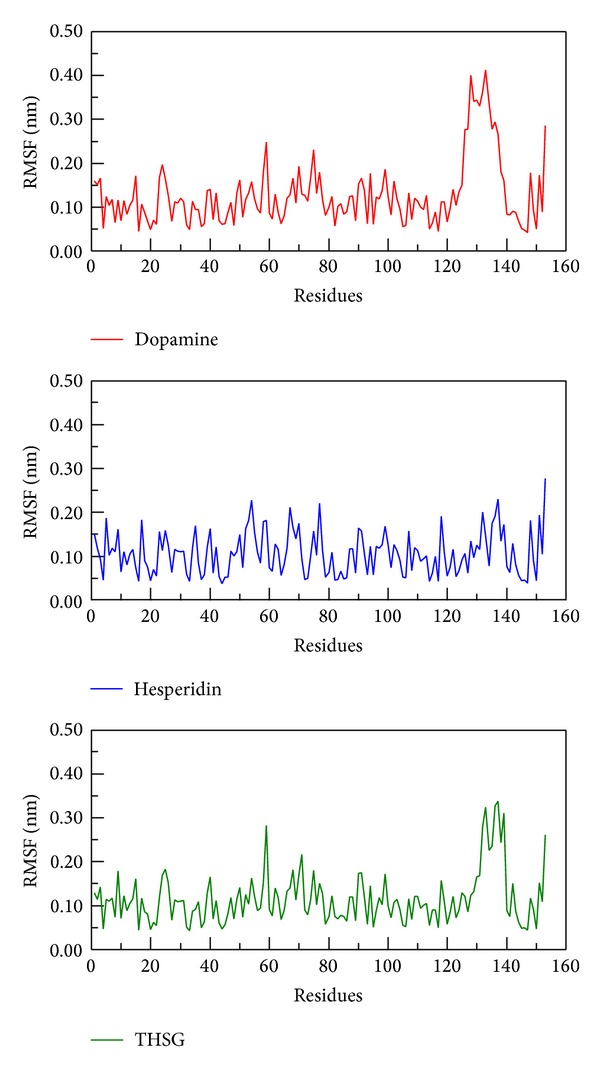
RMSF values of protein residues with docked ligand: dopamine, hesperidin, and THSG among 5000 ps simulation.

**Figure 9:**
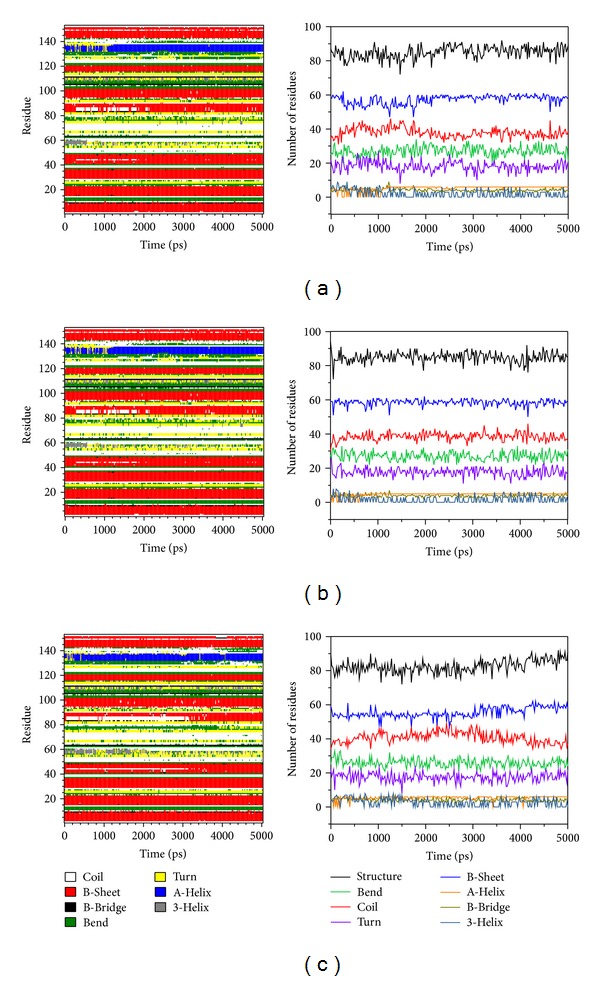
DSSP analysis of complexes with ligands: (a) dopamine (b) hesperidin, and (c) THSG. The “Structure” is summarized by residue number of A-Helix, B-Sheet, B-Bridge, and Turn.

We calculated root mean squared fluctuation (RMSF) to analyze the flexibility of residues on protein structure, and the binding region (from 21 to 32 and from 98 to 100 residues) shows no significant increment on structure of mutant SOD1 with all ligands (Figure 8). From DSSP analysis, all helices and beta-sheets of secondary structure for all complexes continued to exist during 5000 ps simulation times, and the number of residues is not variable among all conformations (Figure 9). The results suggest that the protein structure in each complex remained stable after MD simulations.

### 3.5. Snapshots Analysis

**Figure 10:**
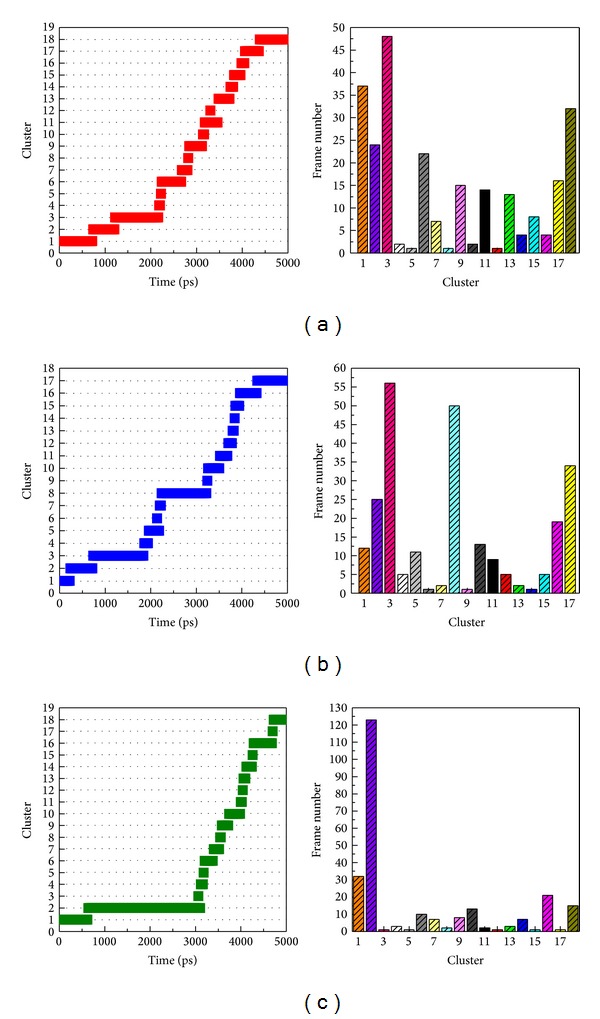
Clustering analyses of all conformations of mutant SOD1 complexes among 5000 ps simulation times.

**Figure 11:**
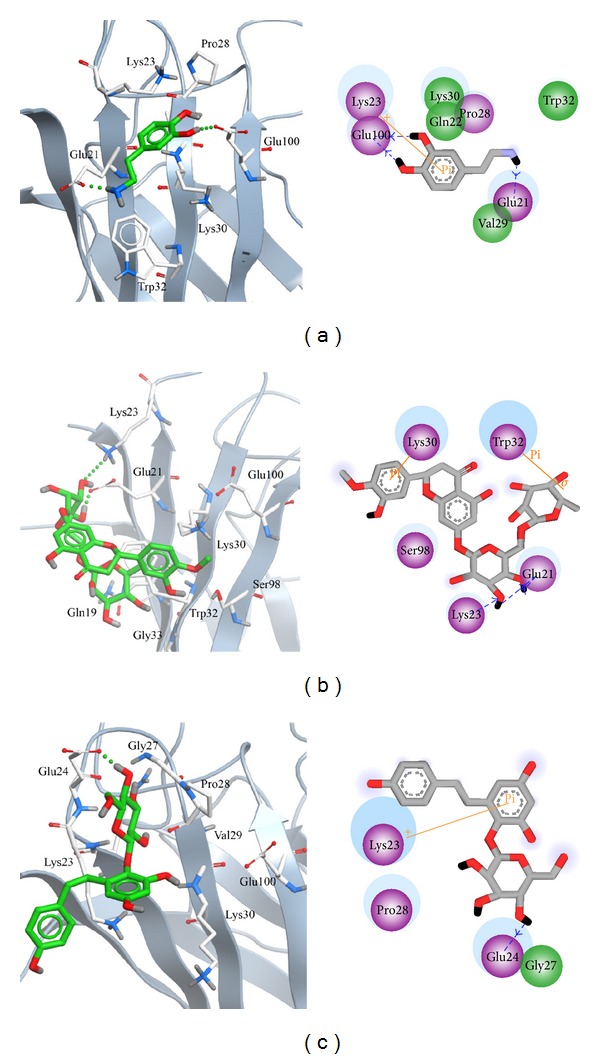
Binding poses of each representative conformation of mutant SOD1 from cluster analyses in 2D and 3D diagrams. Pi interactions (orange), polar residues (purple circle), and van der Waals residues (green circle) are displaced in 2D diagram.

In order to identify the most stable structure during the entire MD simulation for understanding the interaction of docked ligands, all conformations from MD simulation were clustered into seventeen or eighteen groups (Figure 10). We pick up the middle conformation from final groups of clusters as represented structure, and each middle frame is listed in Table 2. In the next study, we analyze protein-ligand interactions of represented structure (Figure 11). Dopamine has H-bond with Glu100 and Glu21, and the pi interaction is formed in Lys23 (Figure 11(a)). Hesperidin generate two H bonds with Lys23 and Glu21; besides, there are two pi interactions displayed on Lys30 and Trp32, respectively (Figure 11(b)). For THSG binding interaction, H bond was found on Glu24 and pi interaction was formed on Lys30 (Figure 11(c)). This result shows that hesperidin and THSG have similar binding residues to dopamine, suggesting that the binding conformations of two candidates are not significantly variable after MD simulations.

## 4. Conclusion

For docking analysis, both Hesperidin and THSG have higher Dock Score than dopamine, and they displayed stable movements for mutant SOD1 from MSD and center mass distance analysis, which was correlated with the low affinity in docking results. For RMSF and DSSP assay, the secondary structure of mutant SOD1 did not change significantly during MD simulation, suggesting that the docked ligands are not affected by protein structure. Hesperidin and THSG have high affinity with SOD1 and the binding interactions are similar to dopamine among all molecular simulations. Our result indicated that hesperidin and THSG might be potential lead compound to design inhibitors of mutant SOD1 for ALS therapy.

## Figures and Tables

**Table 1:** Results of TCM compounds interacted with mutant SOD1 structure by LigandFit docking analysis.

Name	-PLP1	-PLP2	-PMF	DOCK SCORE
Hesperidin	**33.72 **	**39.61 **	**95.25 **	**91.91 **
THSG	**37.36 **	**41.61 **	**101.23 **	**88.16 **
Hyperoside	32.16	41.58	79.96	79.85
Dopamine*	**26.79 **	**30.68 **	**37.71 **	**51.94 **
Nobiletin	44.73	44.01	109.57	41.14
Ursolic acid	36.61	36.50	114.59	34.69
Tangeretin	29.24	32.45	73.20	33.28
Nobiletin	59.41	46.54	117.14	29.82
Lupeol	42.26	42.03	115.03	23.97
Emodin	30.29	32.13	84.07	22.02
Physcion	39.79	37.57	98.34	19.94

*Control.

**Table 2:** Time of middle structure in each cluster among MD simulation times.

Cluster	Time of middle frame (ps)		
	Dopamine	Hesperidin	THSG
1	460	160	300
2	940	360	2080
3	1520	1480	3060
4	2180	1880	3140
5	2220	2040	3180
6	2500	2140	3280
7	2740	2200	3460
8	2820	2920	3540
9	2960	3240	3660
10	3140	3380	3840
11	3380	3580	4000
12	3300	3740	4040
13	3560	3800	4080
14	3780	3840	4160
15	3900	3900	4260
16	4020	4180	4580
17	4280	4620	4700
18	4740	—	4860
